# HIV programmatic outcomes following implementation of the ‘Treat‐All’ policy in a public sector setting in Eswatini: a prospective cohort study

**DOI:** 10.1002/jia2.25458

**Published:** 2020-03-03

**Authors:** Bernhard Kerschberger, Michael Schomaker, Kiran Jobanputra, Serge M Kabore, Roger Teck, Edwin Mabhena, Simangele Mthethwa‐Hleza, Barbara Rusch, Iza Ciglenecki, Andrew Boulle

**Affiliations:** ^1^ Médecins Sans Frontières (Operational Centre Geneva) Mbabane Eswatini; ^2^ Centre for Infectious Disease Epidemiology and Research School of Public Health and Family Medicine University of Cape Town Cape Town South Africa; ^3^ Institute of Public Health, Medical Decision Making and Health Technology Assessment UMIT ‐ University for Health Sciences, Medical Informatics and Technology Hall in Tirol Austria; ^4^ The Manson Unit Médecins Sans Frontières London United Kingdom; ^5^ Eswatini National AIDS Programme (ENAP) Ministry of Health Mbabane Eswatini; ^6^ Médecins Sans Frontières (Operational Centre Geneva) Geneva Switzerland

**Keywords:** treat all, retention, viral failure, Swaziland, Eswatini, HIV

## Abstract

**Introduction:**

The Treat‐All policy – antiretroviral therapy (ART) initiation irrespective of CD4 cell criteria – increases access to treatment. Many ART programmes, however, reported increasing attrition and viral failure during treatment expansion, questioning the programmatic feasibility of Treat‐All in resource‐limited settings. We aimed to describe and compare programmatic outcomes between Treat‐All and standard of care (SOC) in the public sectors of Eswatini.

**Methods:**

This is a prospective cohort study of ≥16‐year‐old HIV‐positive patients initiated on first‐line ART under Treat‐All and SOC in 18 health facilities of the Shiselweni region, from October 2014 to March 2016. SOC followed the CD4 350 and 500 cells/mm^3^ treatment eligibility thresholds. Kaplan‐Meier estimates were used to describe crude programmatic outcomes. Multivariate flexible parametric survival models were built to assess associations of time from ART initiation with the composite unfavourable outcome of all‐cause attrition and viral failure.

**Results:**

Of the 3170 patients, 1888 (59.6%) initiated ART under Treat‐All at a median CD4 cell count of 329 (IQR 168 to 488) cells/mm^3^ compared with 292 (IQR 161 to 430) (*p* < 0.001) under SOC. Although crude programme retention at 36 months tended to be lower under Treat‐All (71%) than SOC (75%) (*p* = 0.002), it was similar in covariate‐adjusted analysis (adjusted hazard ratio [aHR] 1.06, 95% CI 0.91 to 1.23). The hazard of viral suppression was higher for Treat‐All (aHR 1.12, 95% CI 1.01 to 1.23), while the hazard of viral failure was comparable (Treat‐All: aHR 0.89, 95% CI 0.53 to 1.49). Among patients with advanced HIV disease (n = 1080), those under Treat‐All (aHR 1.13, 95% CI 0.88 to 1.44) had a similar risk of an composite unfavourable outcome to SOC. Factors increasing the risk of the composite unfavourable outcome under both interventions were aged 16 to 24 years, being unmarried, anaemia, ART initiation on the same day as HIV care enrolment and CD4 ≤ 100 cells/mm^3^. Under Treat‐All only, the risk of the unfavourable outcome was higher for pregnant women, WHO III/IV clinical stage and elevated creatinine.

**Conclusions:**

Compared to SOC, Treat‐All resulted in comparable retention, improved viral suppression and comparable composite outcomes of retention without viral failure.

## 1 Introduction

The World Health Organization (WHO) recommends antiretroviral therapy (ART) initiation at the time of HIV diagnosis irrespective of clinical and immunological criteria, aiming at improving patient‐level outcomes and reducing HIV transmission 1. High acceptability of this Treat‐All policy led to 93% adoption and 84% implementation coverage in low‐ and middle‐income countries in July 2019 2, and facility‐site level implementation started in southern Africa as early as 2016 3. With an estimated 67% ART coverage in eastern and southern Africa in 2018, an additional three million are required to access treatment with the introduction of Treat‐All 4.

HIV programmes need to maximize retention on and adherence to ART during treatment expansion to take advantage of the clinical, programmatic and public health benefits of Treat‐All and achieve the second and third 90s of the UNAIDS 90‐90‐90 targets 5, 6. However, challenges in ART programming may be exacerbated with Treat‐All, questioning the feasibility of universal ART in resource‐limited settings (RLS) 7, 8, 9, 10, 11, 12, 13, 14, 15. Expanding treatment in overburdened health facilities may overextend health systems and decrease the quality of care, treatment follow‐up and record‐keeping 16, 17, 18. High rates of loss to follow‐up (LTFU), all‐cause attrition and viral failure have been reported from rapidly growing ART cohorts 16, 17, 18, 19, 20, 21, 22, and previous guideline changes to increase the treatment eligibility threshold yielded mixed findings 23, 24. Patients with high CD4 cell counts on ART – a group that will increase with Treat‐All – have a higher risk of unstructured treatment interruptions, missing clinical appointments and LTFU, leading to lower long‐term retention 17, 19, 25, 26, 27. Also, patients should not be de‐prioritized when presenting late to HIV treatment 1, a possible challenge for expanding treatment programmes which may prioritize ART initiations over quality of follow‐up care.

The impact of changing ART eligibility criteria in RLS is poorly understood because of the lack of recent programme data 28, the gap between supporting health policies and efficient operationalization 29, 30 and inconclusive treatment outcome data from ongoing Treat‐All trials 31, 32, 33, 34. Eswatini (formerly Swaziland) is one of the few countries that piloted the Treat‐All policy before it became a WHO recommendation in 2016 1. While Treat‐All has increased timely ART initiation in routine settings 35, 36, 37, studies on longer‐term outcomes are scarce. To inform scale‐up of Treat‐All in RLS, we aimed to assess varying patterns of associations with treatment outcomes under Treat‐All and under the concurrent national standard of care (SOC) at the time, and to compare programmatic outcomes between both interventions.

## 2 Methods

### 2.1 Study design

This is a prospective cohort study of ≥16‐year‐old HIV‐positive patients initiated on first‐line ART under the Treat‐All programmatic approach and under SOC in 18 public sector health facilities of the Shiselweni region (Eswatini), from 20 October 2014 to 31 March 2016.

### 2.2 Study setting

The setting has been described previously 38. The predominantly rural Shiselweni region has a population of ~210,000 39 and HIV prevalence is 31% in 18‐ to 49‐year‐olds 40, 41. The study was conducted in two neighbouring health zones, each comprising eight HIV/TB care integrated primary care facilities and one HIV/TB care collocated secondary care outpatient department. The Treat‐All health zone offered prompt facility‐based ART initiation irrespective of CD4 and clinical criteria for all newly diagnosed patients and those already enrolled in pre‐ART care. The neighbouring SOC health zone followed national treatment guidelines with ART initiation at CD4 ≤ 350 (October 2014 to October 2015) and ≤500 cell/mm^3^ (November 2015 onwards), WHO III/IV clinical staging and the prevention of mother‐to‐child transmission programmatic approach option B+ (PMTCTB+).

Trained lay counsellors conducted HIV testing, and pre‐treatment and treatment adherence counselling. ART initiation and follow‐up care were performed by nurses in primary care clinics and supported by onsite medical doctors in secondary care outpatient departments. Patients usually had a baseline CD4 cell count and laboratory test (haemoglobin, alanine aminotransferase (ALT), creatinine). The CD4 result was not a requirement for ART initiation under Treat‐All. Routine viral load (VL) monitoring was available (using the Biocentric platform 42, 43) with VL testing recommended at six and twelve months after ART initiation, and annually thereafter 43. Enhanced adherence counselling was provided for patients with a VL ≥ 1000 copies/mL, with treatment switching in case of viral failure (two consecutive VLs ≥ 1000 copies/mL). Telephonic and physical defaulter tracing was recommended for patients missing clinical appointments.

### 2.3 Analyses, outcomes and definitions

**Figure 1:**
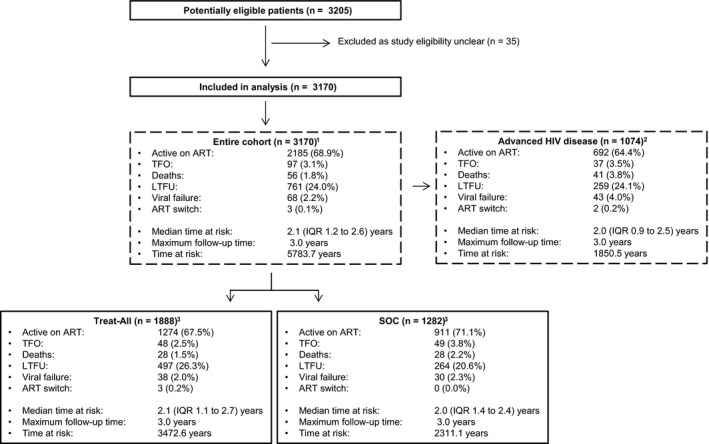
Study flow and analyses performed. ^1^The analysis directly compares Treat‐All with SOC irrespective of CD4 and WHO clinical staging criteria. ^2^The analysis directly compares Treat‐All with SOC restricted to patients with advanced HIV disease (CD<200 cells/mm^3^ and/or WHO III/IV). ^3^The treat‐All and SOC interventions were analysed separately. ART, antiretroviral therapy; n, number; IQR, interquartile range; LTFU, loss to follow‐up; SOC, standard of care; TFO, transferred out.

Several analyses were conducted (Figure 1). First, baseline factors were described separately for both interventions. Laboratory measures were recorded at the time of ART initiation and a TB case was defined as a patient receiving TB treatment between six months before and three months after ART initiation. Calendar time was divided into time period‐1 and time period‐2, corresponding to the WHO 2010 (October 2014 to October 2015) and WHO 2013 (November 2015 onwards) treatment guideline implementation periods followed under SOC. Same‐day ART initiation was defined as patients starting ART on the same day as HIV care enrolment (the date of opening a patient file at the health facility).

Second, we describe crude and covariate adjusted programmatic indicators. Retention was defined as patients in ART care at different time points (without the outcome of death or LTFU). We chose this end point because the vital status was not actively ascertained in both interventions. LTFU was defined as six months without a clinic visit measured from the last clinic visit. Follow‐up time was censored at database closure (31 October 2017) or date of transfer out of the facility. Then we describe VL testing uptake (the probability of receiving at least one VL test) and viral suppression, defined as the proportion of VLs < 1000 copies/mL among patients with a first VL measurement recorded. Finally, viral failure was compared, defined as two consecutive VLs ≥ 1000 copies/mL measured at least five months after ART initiation and performed ≥1.5 months apart or treatment switching to a protease inhibitor based regimen with two new drugs in the absence of documented viral failure. All viral load outcomes were measured from five months after ART initiation.

Third, Treat‐All aims to retain patients on virally suppressed ART to improve patient level outcomes and reduce transmission of HIV. Accordingly, we established a composite primary endpoint of death, LTFU and viral failure. To assess whether the unfavourable outcome was more likely to occur in the Treat‐All zone, we compared the interventions directly, first for the entire cohort and then restricted to patients presenting with advanced HIV disease defined as CD4 < 200 cells/mm^3^ and/or WHO III/IV clinical staging.

Finally, we conducted separate analyses of the composite primary endpoint for both models of care to assess possible varying patterns of associations with Treat‐All and SOC during the implementation of different treatment guidelines.

All data were collected by trained data clerks from individual‐level clinic records and paper registers, and entered into EpiData software. VL and TB data were complemented with data from separate electronic databases used for routine programme monitoring.

### 2.4 Statistics

Baseline characteristics and crude programmatic outcomes were described with frequencies and proportions, and compared using the Pearson's chi‐squared test for categorical variables and Wilcoxon's rank‐sum test for continuous variables. Kaplan‐Meier estimates were used to describe retention, VL testing uptake and viral failure.

Variables for inclusion in multivariate analyses were selected a priori based on clinical relevance and literature review. We used multiple imputation by chained equations 44 to impute missing covariate data using 20 datasets (Table S1). Multiple imputation diagnostics were satisfied according to trace plots and Kernel density plots (Figure S1 and S2). Covariate adjusted parametric survival models (Royston‐Parmar models) 45 were built to describe associations with time to the composite outcome. We used Akaike's information criteria to determine the number and location of internal knots for the baseline spline function. Covariates violating the proportional hazards assumption (assessed with Schoenfeld residual statistics) were included in the models as time‐varying effects. All analyses were performed with Stata 14.1 (StataCorp, College Station, Texas, USA).

### 2.5 Ethics

This study was approved by the Scientific and Ethics Committee of Eswatini, the Research Ethics Committees of MSF and the University of Cape Town, South Africa. Informed written consent was obtained before ART initiation from patients in Treat‐All who were ineligible for ART according to the national SOC.

## 3 Results

### 3.1 Baseline characteristics

#### 3.1.1 Treat‐All and SOC

Figure 1 shows the study flow. Thirty‐five patients were removed from the analysis because study eligibility was unclear. Of the remaining 3170 patients (Table 1a), 1888 (59.6%) initiated treatment under Treat‐All. Baseline factors comparable between health zones were attendance at primary care level (64.5%), being pregnant (22.7%), median age (31 years, interquartile range (IQR) 25 to 38), clinical factors (body mass index (BMI), haemoglobin, ALT, creatinine) and TB co‐infection (7.0%). Under Treat‐All, patients were more likely to receive tenofovir (TDF) (98.7% vs. 92.6%) and efavirenz (EFV) (98.9% vs. 92.4%) based ART regimens, start treatment on the same day as HIV diagnosis (22.8% vs. 13.9%) and HIV care enrolment (44.4% vs. 24.3%), have secondary education (67.7% vs. 57.7%) and not have access to a phone (11.4% vs. 7.9%). Overall, patients had a higher median CD4 cell count under Treat‐All (329, IQR 168 to 488, vs. 292, 161 to 430; *p* < 0.001). The difference was highest for non‐pregnant adults in period‐1 when SOC applied the CD4 ≤ 350 treatment eligibility threshold (334, IQR 156 to 494, vs. 260.5, IQR 124 to 362; *p* < 0.001).

**Table 1:** Baseline characteristics of patients initiating first‐line ART under Treat‐All and standard of care (SOC) (entire treatment cohort and patients with advanced HIV disease)

(Missing values for entire cohort)	(a) Entire cohort (n = 3170)			(b) Advanced HIV disease (n = 1074)^a^		
	Treat‐All (n = 1888) (n, %)	SOC (n = 1282) (n, %)	*p* value	Treat‐All (n = 620) (n, %)	SOC (n = 454) (n, %)	*p* value
Implementation period^b^						
Period‐1	1397 (74.0)	851 (66.4)	<0.001	428 (69.0)	317 (69.8)	0.781
Period‐2	491 (26.0)	431 (33.6)		192 (31.0)	137 (30.2)	
Facility						
PHC	1215 (64.4)	830 (64.7)	0.822	394 (63.5)	249 (54.8)	0.004
SHC^c^	673 (35.6)	452 (35.3)		226 (36.5)	205 (45.2)	
Time since HIV diagnosis (0.5%)^d^						
≥90 days	572 (30.5)	499 (39.1)	<0.001	119 (19.3)	114 (25.2)	<0.001
1 to 89 days	877 (46.7)	601 (47.1)		376 (61.0)	305 (67.3)	
Same day	429 (22.8)	177 (13.9)		121 (19.6)	34 (7.5)	
Time since HIV care enrolment^e^						
≥90 days	308 (16.3)	325 (25.4)	<0.001	36 (5.8)	44 (9.7)	<0.001
1 to 89 days	741 (39.2)	646 (50.4)		321 (51.8)	331 (72.9)	
Same day	839 (44.4)	311 (24.3)		263 (42.4)	79 (17.4)	
Sex						
Men	510 (27.0)	400 (31.2)	0.011	262 (42.3)	221 (48.7)	0.037
Women	1378 (73.0)	882 (68.8)		358 (57.7)	233 (51.3)	
Pregnancy (0.5%)						
No	1443 (76.8)	996 (78.2)	0.349	540 (87.5)	421 (92.7)	0.006
Yes	437 (23.2)	278 (21.8)		77 (12.5)	33 (7.3)	
Age at HIV care enrolment, years						
16 to 24	416 (22.0)	292 (22.8)	0.877	71 (11.5)	43 (9.5)	0.391
25 to 49	1317 (69.8)	884 (69.0)		486 (78.4)	356 (78.4)	
≥50	155 (8.2)	106 (8.3)		63 (10.2)	55 (12.1)	
Marital status (1.4%)						
Married	638 (34.5)	594 (46.6)	<0.001	240 (39.6)	227 (50.1)	0.001
Not married	1212 (65.5)	681 (53.4)		366 (60.4)	226 (49.9)	
Education (14.4%)						
None	75 (4.8)	101 (8.9)	<0.001	34 (6.6)	44 (11.0)	0.105
Primary	410 (26.0)	359 (31.6)		145 (28.0)	114 (28.5)	
Secondary	1068 (67.7)	656 (57.7)		330 (63.7)	236 (59.0)	
Tertiary	25 (1.6)	21 (1.8)		9 (1.7)	6 (1.5)	
CD4 count, cells/mm^3^ (3.0%)						
0 to 100	277 (15.1)	192 (15.4)	<0.001	277 (45.3)	192 (42.3)	0.295
101 to 200	274 (15.0)	200 (16.1)		272 (44.4)	200 (44.1)	
201 to 350	424 (23.2)	387 (31.1)		31 (5.1)	37 (8.1)	
351 to 500	420 (23.0)	273 (21.9)		15 (2.5)	14 (3.1)	
≥501	434 (23.7)	194 (15.6)		17 (2.8)	11 (2.4)	
WHO clinical stage (0.5%)						
I	1282 (68.3)	904 (70.7)	<0.001	255 (41.3)	182 (40.4)	0.028
II	365 (19.5)	177 (13.8)		133 (21.6)	72 (16.0)	
III/IV	229 (12.2)	197 (15.4)		229 (37.1)	197 (43.7)	
Tuberculosis						
No	1763 (93.4)	1186 (92.5)	0.347	520 (83.9)	384 (84.6)	0.753
Yes	125 (6.6)	96 (7.5)		100 (16.1)	70 (15.4)	
BMI, kg/m^2^ (6.3%)						
<18.5	105 (6.1)	81 (6.5)	0.113	70 (12.3)	60 (13.8)	0.739
18.5 to 24.9	869 (50.1)	670 (53.6)		343 (60.3)	254 (58.3)	
≥25	759 (43.8)	500 (40.0)		156 (27.4)	122 (28.0)	
Haemoglobin, g/dL (22.6%)						
≤9	262 (18.1)	156 (15.6)	0.102	122 (25.8)	89 (24.5)	0.645
≥10	1187 (81.9)	847 (84.4)		350 (74.2)	275 (75.5)	
ALT, U/L (30.1%)						
≤42	1289 (87.4)	639 (86.2)	0.446	393 (82.0)	213 (81.8)	0.883
≥43	186 (12.6)	102 (13.8)		86 (18.0)	48 (18.4)	
Creatinine, µmol/L (20.6%)						
≤120	1532 (97.7)	926 (97.7)	0.968	484 (95.5)	315 (94.6)	0.568
≥121	36 (2.3)	22 (2.3)		23 (4.5)	18 (5.4)	
NRTI						
TDF	1864 (98.7)	1187 (92.6)	<0.001	610 (98.4)	410 (90.3)	<0.001
AZT	20 (1.1)	83 (6.5)		8 (1.3)	34 (7.5)	
ABC	4 (0.2)	12 (0.9)		2 (0.3)	10 (2.2)	
NNRTI						
EFV	1868 (98.9)	1184 (92.4)	<0.001	614 (99.0)	417 (91.9)	<0.001
NVP	20 (1.1)	98 (7.6)		6 (1.0)	37 (8.1)	
Phone availability (1.0%)						
No	213 (11.4)	100 (7.9)	0.001	76 (12.3)	28 (6.2)	0.001
Yes	1652 (88.6)	1172 (92.1)		541 (87.7)	423 (93.8)	

ABC, abacavir; ALT, alanine aminotransferase; AZT, zidovudine; BMI, body mass index; EFV, efavirenz; IQR, interquartile range; n, number; NNRTI, non‐nucleoside reverse transcriptase inhibitors; NRTI, nucleoside reverse transcriptase inhibitor; NVP, nevirapine; PHC, primary healthcare level; SHC, secondary healthcare level; SOC, standard of care; TDF, tenofovir disoproxil fumarate; WHO, World Health Organization.

a Advanced HIV disease was defined as patients presenting with CD4 < 200 cells/mm^3^ and/or WHO III/IV staging;

b period‐1 is the WHO 2010 (from October 2014 to October 2015) and period‐2 is the WHO 2013 (from November 2015 onwards) ART initiation guideline implementation period as followed under standard of care;

c secondary healthcare: ART outpatient departments in one health centre (with inpatient capacity) in Treat‐All and ART outpatient departments in one hospital in standard of care;

d this is the time from HIV diagnosis to ART initiation;

e this is the time from facility‐based HIV care enrolment to ART initiation.

#### 3.1.2 Advanced HIV disease

Overall, 1074/3170 (33.9%) patients presented with advanced HIV disease, of whom 620 (57.8%) were under Treat‐All. Distribution of baseline factors is presented in Table 1b. Of the patients with available CD4 cell count and WHO clinical staging data, 642/1060 (60.6%) presented with both CD4 cell count ≤200 cells/mm^3^ and WHO clinical stage I/II (Table S2).

### 3.2 Programmatic outcomes

The frequency of the outcomes is presented in Figure 1.

#### 3.2.1 Retention

##### Treat‐all

**Figure 2:**
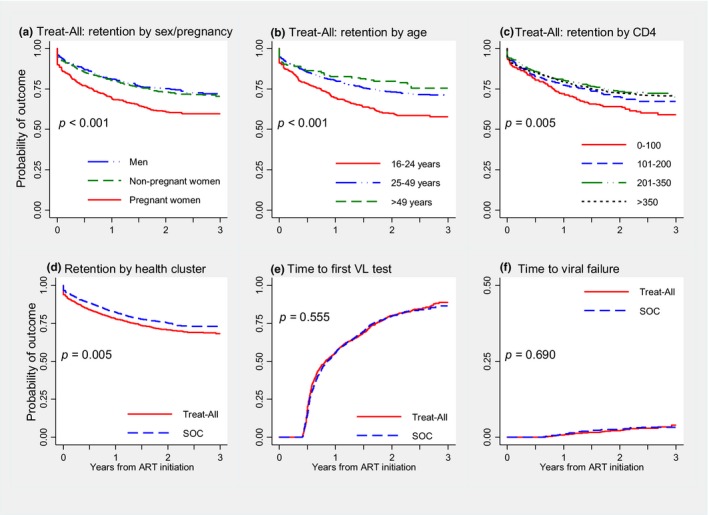
Kaplan‐Meier plots of retention on antiretroviral therapy for different baseline characteristics under Treat‐All (**a‐c**), for Treat‐All versus SOC (entire cohort) (**d**), and for time to first viral test uptake (**e**) and time to viral failure (**f**). Viral load (VL) uptake and viral failure analyses: Observation time started five months after ART initiation for patients without unfavourable outcome until five months after ART initiation. Patients were right censored in case of loss to care, deaths, transfer out or without a VL test at the end of the observation period. ART, antiretroviral therapy; SOC, standard of care; VL, viral load.

Under Treat‐All, pregnant women (vs. men, non‐pregnant women) (*p* < 0.001), younger patients (16 to 24 years) (*p* < 0.001) and those with low CD4 cell count (≤100 cells/mm^3^) (*p* = 0.005) tended to have had lower retention (Figure 2a‐c, Table S3). Additional Kaplan‐Meier graphs of retention under Treat‐All are in Figure S3.

##### Treat‐all versus SOC

**Figure 3:**
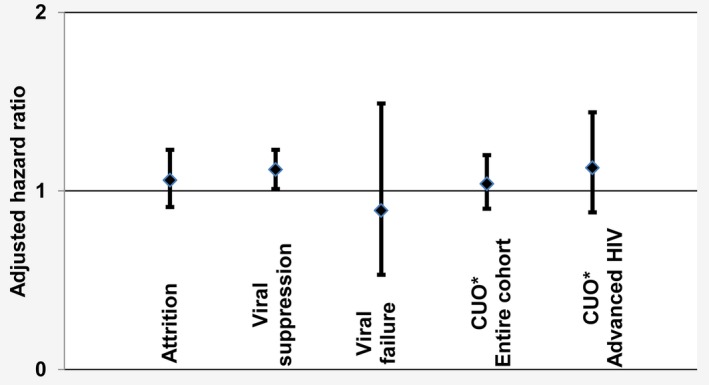
Summary of covariate adjusted hazard ratios of different programmatic outcomes under Treat‐All when compared with standard of care (zero line). CUO, composite unfavourable outcome.

Comparing both health zones, crude 6‐ and 36‐month retention were 84% and 71% under Treat‐All compared with 89% and 75% under SOC (*p* = 0.005) (Figure 2d). Retention tended to be lower for pregnant women than non‐pregnant adults under both interventions (Table 2). Overall, 6% and 3% of patients under Treat‐All and SOC never came for a follow‐up visit after initiation of ART. In covariate‐adjusted analysis, the hazard of attrition was similar for Treat‐all (adjusted hazard ratio (aHR) 1.06, 95% CI 0.91 to 1.23) compared with SOC (*model not shown*) (Figure 3).

**Table 2:** Kaplan–Meier estimates of retention on antiretroviral therapy, overall and for pregnant and non‐pregnant adults (percentage and 95% confidence interval)

	Entire cohort (n = 3170)		Advanced HIV disease (n = 1074)	
	Treat‐All (n = 1888)	SOC (n = 1282)	Treat‐All (n = 620)	SOC (n = 454)
Retention, entire cohort				
1 day^a^	94 (93 to 95)	97 (95 to 97)	95 (93 to 96)	95 (93 to 97)
6 months	84 (82 to 85)	89 (87 to 90)	81 (78 to 84)	86 (82 to 89)
12 months	78 (76 to 80)	83 (81 to 85)	76 (73 to 80)	81 (77 to 84)
24 months	72 (70 to 74)	77 (75 to 79)	70 (66 to 73)	74 (70 to 78)
36 months	71 (69 to 73)	75 (73 to 78)	68 (64 to 71)	72 (68 to 76)
Retention, non‐pregnant adults				
1 day^a^	95 (94 to 96)	97 (96 to 98)	96 (94 to 97)	95 (92 to 97)
6 months	86 (84 to 88)	90 (88 to 92)	82 (78 to 85)	86 (82 to 89)
12 months	81 (79 to 83)	85 (83 to 87)	76 (72 to 80)	81 (77 to 84)
24 months	75 (73 to 77)	79 (77 to 82)	70 (66 to 74)	75 (70 to 79)
36 months	74 (72 to 76)	78 (75 to 80)	68 (64 to 72)	72 (67 to 76)
Retention, pregnant women				
1 day^a^	90 (87 to 93)	94 (90 to 96)	87 (77 to 93)	97 (80 to 100)
6 months	77 (73 to 81)	84 (79 to 88)	79 (68 to 87)	85 (67 to 93)
12 months	70 (65 to 74)	77 (71 to 81)	78 (67 to 86)	75 (56 to 87)
24 months	62 (57 to 66)	70 (64 to 75)	69 (57 to 78)	72 (53 to 84)
36 months	61 (56 to 65)	67 (61 to 73)	65 (53 to 75)	72 (53 to 84)

SOC, standard of care.

a These patients never came back for a clinic visit after ART initiation.

#### 3.2.2 Viral outcomes

Among patients retained in care for ≥5 months (Treat‐All: n = 1587, SOC: n = 1127), time to first VL test was similarly low and delayed in both health zones at 6 (Treat‐All: 19%, SOC: 15%) and 36 months (Treat‐All: 89%, SOC: 86%) (*p* = 0.555) (Figure 2e), with 90% and 87% respectively being virally supressed (*p* = 0.012). In multivariate analysis, the hazard of viral suppression was higher for Treat‐all (aHR 1.12, 95% CI 1.01 to 1.23) than SOC (*model not shown*) (Figure 3). Assuming that patients without a VL test result and retained in care ≥5 months had an elevated VL, viral suppression decreased to 76% under Treat‐All and 72% under SOC (*p* = 0.019). The cumulative 3‐year hazard of viral failure was 4% under Treat‐All and 3% under SOC (*p* = 0.690) (Figure 2f) and remained comparable in covariate‐adjusted analysis (Treat‐All: aHR 0.89, 95% CI 0.53 to 1.49) (*model not shown*) (Figure 3).

#### 3.2.3 Composite unfavourable outcome (death, LTFU and viral failure)

Comparing Treat‐All and SOC in one covariate‐adjusted model, the hazard of the unfavourable outcome was similar for the entire cohort (Treat‐All: aHR 1.04, 95% CI 0.90 to 1.20) (Table S4) and for patients with advanced HIV disease (Treat‐All: aHR 1.13, 95% CI 0.88 to 1.44) (Table 3, Figure 3).

**Table 3:** Predictors of the unfavourable outcome for patients with advanced HIV disease (Treat‐All and SOC combined) initiated on first‐line ART (n = 1080)

	Univariate analysis	Multivariate analysis^a^
	HR (95% CI)	aHR (95% CI)
Health zone		
SOC	1	1
Treat‐All	1.15 (0.93 to 1.43)	1.13 (0.88 to 1.44)
Implementation period^b^		
Period‐1	1	1
Period‐2	0.87 (0.68 to 1.10)	0.83 (0.64 to 1.07)
Facility		
PHC	1	1
SHC^c^	0.88 (0.71 to 1.10)	0.84 (0.65 to 1.07)
Time since HIV diagnosis^d^		
≥90 days	1	1
1 to 89 days	0.80 (0.63 to 1.04)	0.86 (0.62 to 1.18)
Same day	0.94 (0.67 to 1.33)	0.65 (0.42 to 1.00)
Time since HIV care enrolment^e^		
≥90 days	1	1
1 to 89 days	0.73 (0.50 to 1.07)	0.84 (0.53 to 1.36)
Same day	1.00 (0.67 to 1.48)	1.21 (0.75 to 1.96)
Sex		
Men	1	1
Women	1.29 (1.04 to 1.60)	1.17 (0.91 to 1.51)
Pregnancy		
No	1	1
Yes	1.07 (0.76 to 1.51)	1.03 (0.69 to 1.53)
Age at HIV care enrolment, years		
16 to 24	1.46 (1.08 to 1.99)	1.22 (0.87 to 1.72)
25 to 49	1	1
≥50	0.91 (0.64 to 1.31)	1.08 (0.74 to 1.59)
Marital status		
Married	1	1
Not married	1.76 (1.40 to 2.21)	1.53 (1.19 to 1.97)
Education		
None	1	1
Primary	1.11 (0.71 to 1.73)	1.08 (0.68 to 1.71)
Secondary	0.99 (0.65 to 1.51)	0.97 (0.62 to 1.51)
Tertiary	1.16 (0.46 to 2.91)	1.10 (0.41 to 2.91)
CD4 count, cells/mm^3^		
0 to 100	1.39 (0.85 to 2.26)	1.59 (0.94 to 2.68)
101 to 200	1.05 (0.64 to 1.72)	1.35 (0.78 to 2.33)
201 to 350	1	1
≥351	0.98 (0.50 to 1.93)	1.05 (0.52 to 2.12)
WHO clinical stage		
I	1	1
II	1.10 (0.82 to 1.48)	1.03 (0.75 to 1.41)
III/IV	1.20 (0.94 to 1.51)	1.22 (0.91 to 1.66)
Tuberculosis		
No	1	1
Yes	0.94 (0.71 to 1.26)	0.76 (0.56 to 1.04)
BMI, kg/m^2^		
<18.5	1.87 (1.42 to 2.47)	1.89 (1.40 to 2.54)
18.5 to 24.9	1	1
≥25	0.87 (0.67 to 1.14)	0.94 (0.70 to 1.26)
Haemoglobin, g/dL		
≤9	1.63 (1.28 to 2.08)	1.43 (1.09 to 1.87)
≥10	1	1
ALT, U/L		
≤42	1	1
≥43	1.24 (1.01 to 1.54)	1.03 (0.80 to 1.33)
Creatinine, µmol/L		
≤120	1	1
≥121	1.67 (1.33 to 2.09)	1.77 (1.35 to 2.32)
NRTI		
TDF	1	1
AZT	1.29 (0.78 to 2.12)	1.03 (0.47 to 2.26)
ABC	1.12 (0.42 to 3.01)	0.95 (0.34 to 2.67)
NNRTI		
EFV	1	1
NVP	1.25 (0.76 to 2.07)	1.42 (0.65 to 3.13)
Phone availability		
No	1	1
Yes	0.80 (0.57 to 1.12)	1.10 (0.76 to 1.60)

ABC, abacavir; aHR, adjusted hazard ratio; ALT, alanine aminotransferase; AZT, zidovudine; BMI, body mass index; CI, confidence interval; EFV, efavirenz; HR, hazard ratio; NNRTI, non‐nucleoside reverse transcriptase inhibitors; NRTI, nucleoside reverse transcriptase inhibitor; NVP, nevirapine; PHC, primary healthcare level; SHC, secondary healthcare level; SOC, standard of care; TDF, tenofovir disoproxil fumarate; WHO, World Health Organization.

a The proportional hazard assumption was satisfied. The flexible parametric model had three internal knots;

b period‐1 is the WHO 2010 (from October 2014 to October 2015) and period‐2 is the WHO 2013 (from November 2015 onwards) ART initiation guideline implementation period as followed under standard of care;

c secondary healthcare: ART outpatient departments in one health centre (with inpatient capacity) in Treat‐All and ART outpatient departments in one hospital in standard of care;

d this is the time from HIV diagnosis to ART initiation;

e this is the time from facility‐based HIV care enrolment to ART initiation.

In both analyses, the hazard was higher for BMI < 18.5 kg/m^2^, haemoglobin ≤9 g/dL, creatinine ≥121 µmol/L and being unmarried. The hazard was higher for the entire cohort only for the CD4 strata ≤200 cells/mm^3^ (vs. CD4 201 to 350), WHO clinical stage III/IV (vs. WHO stage I), age 16 to 24 years (vs. 25 to 49 years) and shorter time since HIV care enrolment. The hazard of the unfavourable outcome varied over time for TB, with lower hazard during the first nine months after ART initiation and similar hazard thereafter (Figure S4).

### 3.3 Predictors of the composite unfavourable outcome (Treat‐All vs. SOC)

**Figure 4:**
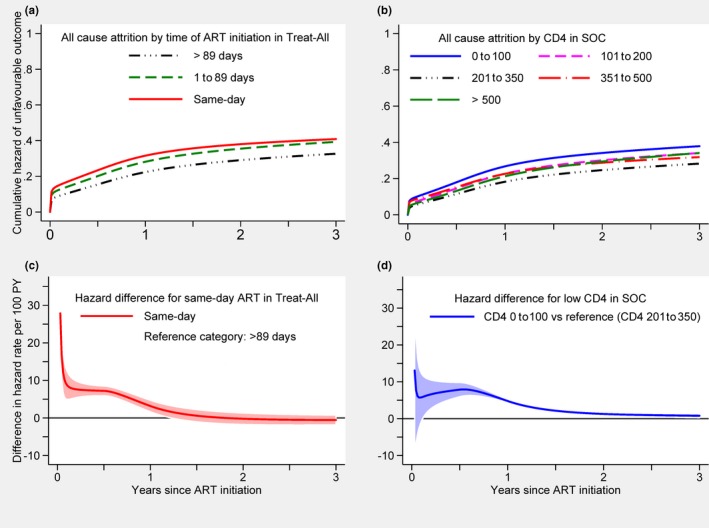
Cumulative hazard and absolute difference in hazard of an unfavourable outcome by time since HIV care enrolment under Treat‐All (**a,c**) and by CD4 cell strata under standard of care (**b,d**) for all patients. ART, antiretroviral therapy; PY, person years; SOC, standard of care.

A breakdown of crude outcomes is shown in Figure 1 and predictors in Table 4. In both health zones, the hazard of the unfavourable outcome was higher for young adults aged 16 to 24 years (vs. 25 to 49 years), unmarried patients, haemoglobin ≤9 g/dL, ART initiation on the same day or within three months of HIV care enrolment (vs. ART initiation after three months) and CD4 cell count ≤100 cells/mm^3^. The effect of same‐day ART initiation varied over time under Treat‐All, with higher hazards during the first 1.1 years after ART initiation while the difference in hazard ceased thereafter (Figure 4a,c). The effect of baseline CD4 varied over time under SOC, with the highest hazard during the first year of treatment for CD4 ≤ 100 cells/mm^3^ (vs. CD4 201 to 350) (Figure 4b,d). Although the hazard difference decreased thereafter, it remained higher for almost the entire observation period. Other factors did not show any strong associations.

**Table 4:** Predictors of the unfavourable outcome for patients initiated on first‐line ART under Treat‐All and standard of care

	Treat‐All (n = 1888)		SOC (n = 1282)	
	Univariate analysis	Multivariate analysis^a^	Univariate analysis	Multivariate analysis^b^
	HR (95% CI)	aHR (95% CI)	HR (95% CI)	aHR (95% CI)
Implementation period^c^				
Period‐1	1	1	1	1
Period‐2	1.34 (1.12 to 1.62)	1.12 (0.92 to 1.36)	0.70 (0.55 to 0.91)	0.71 (0.54 to 0.92)
Facility				
PHC	1	1	1	1
SHC^d^	0.93 (0.78 to 1.11)	0.87 (0.72 to 1.05)	1.01 (0.81 to 1.28)	0.94 (0.72 to 1.23)
Time since HIV diagnosis^a^				
≥90 days	1	1	1	1
1 to 89 days	1.51 (1.22 to 1.85)	1.12 (0.86 to 1.46)	1.72 (1.33 to 2.23)	1.05 (0.74 to 1.48)
Same day	1.76 (1.39 to 2.23)	0.98 (0.73 to 1.30)	2.52 (1.83 to 3.46)	0.92 (0.59 to 1.44)
Time since HIV care enrolment^e^				
≥90 days	1	1	1	1
1 to 89 days	1.99 (1.47 to 2.69)	1.53 (1.02 to 2.30)	2.29 (1.62 to 3.24)	2.07 (1.30 to 3.31)
Same day	2.62 (1.95 to 3.52)	2.08 (1.42 to 3.06)	3.71 (2.59 to 5.32)	3.11 (1.91 to 5.05)
Sex				
Men	1	1	1	1
Women	1.25 (1.03 to 1.52)	1.14 (0.90 to 1.45)	1.18 (0.92 to 1.50)	1.03 (0.76 to 1.39)
Pregnancy				
No	1	1	1	1
Yes	1.62 (1.36 to 1.94)	1.37 (1.10 to 1.71)	1.51 (1.19 to 1.93)	1.21 (0.88 to 1.68)
Age at HIV care enrolment, years				
16 to 24	1.62 (1.35 to 1.95)	1.32 (1.08 to 1.63)	1.83 (1.45 to 2.32)	1.48 (1.11 to 1.98)
25 to 49	1	1	1	1
≥50	0.81 (0.58 to 1.14)	1.04 (0.72 to 1.50)	0.88 (0.56 to 1.39)	1.02 (0.64 to 1.64)
Marital status				
Married	1	1	1	1
Not married	1.70 (1.40 to 2.06)	1.48 (1.20 to 1.81)	1.62 (1.29 to 2.03)	1.39 (1.09 to 1.78)
Education				
None	1	1	1	1
Primary	1.03 (0.62 to 1.70)	1.05 (0.62 to 1.77)	1.05 (0.68 to 1.64)	1.01 (0.64 to 1.60)
Secondary	1.30 (0.80 to 2.10)	1.15 (0.68 to 1.94)	0.99 (0.65 to 1.52)	0.87 (0.56 to 1.37)
Tertiary	1.54 (0.70 to 3.40)	1.23 (0.53 to 2.83)	1.81 (0.82 to 4.00)	2.15 (0.92 to 5.04)
CD4 count, cells/mm^3^				
0 to 100	1.48 (1.14 to 1.93)	1.48 (1.10 to 1.98)	1.93 (1.39 to 2.69)	1.75 (1.19 to 2.55)
101 to 200	1.15 (0.87 to 1.53)	1.19 (0.89 to 1.59)	1.43 (1.01 to 2.03)	1.36 (0.94 to 1.99)
201 to 350	1	1	1	1
351 to 500	1.05 (0.81 to 1.35)	1.16 (0.89 to 1.50)	1.18 (0.84 to 1.66)	1.37 (0.95 to 1.97)
≥501	1.04 (0.81 to 1.34)	1.18 (0.91 to 1.53)	1.44 (1.01 to 2.05)	1.31 (0.87 to 1.96)
WHO clinical stage				
I	1	1	1	1
II	0.86 (0.69 to 1.08)	0.96 (0.75 to 1.22)	1.30 (0.96 to 1.76)	1.16 (0.84 to 1.59)
III/IV	1.34 (1.07 to 1.70)	1.41 (1.05 to 1.90)	1.39 (1.04 to 1.85)	1.07 (0.74 to 1.54)
Tuberculosis				
No	1	1	1	1
Yes	0.91 (0.65 to 1.28)	0.72 (0.49 to 1.04)	1.01 (0.67 to 1.52)	0.68 (0.43 to 1.08)
BMI, kg/m^2^				
<18.5	1.34 (0.98 to 1.85)	1.31 (0.93 to 1.84)	2.42 (1.72 to 3.41)	2.21 (1.52 to 3.21)
18.5 to 24.9	1	1	1	1
≥25	0.84 (0.70 to 1.01)	0.87 (0.72 to 1.07)	0.91 (0.72 to 1.16)	0.89 (0.68 to 1.16)
Haemoglobin, g/dL				
≤9	1.65 (1.34 to 2.03)	1.32 (1.06 to 1.65)	1.81 (1.38 to 2.39)	1.39 (1.02 to 1.90)
≥10	1	1	1	1
ALT, U/L				
≤42	1	1	1	1
≥43	0.84 (0.62 to 1.13)	0.86 (0.63 to 1.17)	0.78 (0.51 to 1.20)	0.81 (0.51 to 1.30)
Creatinine, µmol/L				
≤120	1	1	1	1
≥121	1.65 (0.99 to 2.75)	1.73 (1.00 to 2.99)	1.67 (0.81 to 3.42)	1.95 (0.91 to 4.17)
NRTI				
TDF	1	1	1	1
AZT	0.64 (0.24 to 1.71)	0.67 (0.17 to 2.66)	1.24 (0.82 to 1.86)	1.23 (0.64 to 2.38)
ABC	3.10 (1.00 to 9.65)	2.96 (0.89 to 9.91)	1.34 (0.50 to 3.60)	1.06 (0.38 to 2.96)
NNRTI				
EFV	1	1	1	1
NVP	0.63 (0.23 to 1.68)	0.66 (0.17 to 2.58)	1.12 (0.75 to 1.66)	1.13 (0.61 to 2.11)
Phone availability				
No	1	1	1	1
Yes	0.89 (0.69 to 1.15)	0.80 (0.61 to 1.06)	1.06 (0.69 to 1.62)	1.09 (0.70 to 1.70)

ABC, abacavir; aHR, adjusted hazard ratio; ALT, alanine aminotransferase; AZT, zidovudine; BMI, body mass index; CI, confidence interval; EFV, efavirenz; HR, hazard ratio; NNRTI, non‐nucleoside reverse transcriptase inhibitors; NRTI, nucleoside reverse transcriptase inhibitor; NVP, nevirapine; PHC, primary healthcare level; SHC, secondary healthcare level; SOC, standard of care; TDF, tenofovir disoproxil fumarate; WHO, World Health Organization.

a This is the time from HIV diagnosis to ART initiation;

b specifications of the flexible parametric models: Treat‐All: three main internal knots and two internal knots for the time‐varying covariate (time since HIV care enrolment). SOC: three main internal knots and one internal knot for the time‐varying covariate (CD4 cell count);

c period‐1 is the WHO 2010 (from October 2014 to October 2015) and period‐2 is the WHO 2013 (from November 2015 onwards) ART initiation guideline implementation period as followed under standard of care;

d secondary healthcare: ART outpatient departments in one health centre (with inpatient capacity) in Treat‐All and ART outpatient departments in one hospital in standard of care;

e this is the time from facility‐based HIV care enrolment to ART initiation.

Under SOC only, the hazard was higher for low BMI < 18.5 kg/m^2^ (aHR 2.21, 95% CI 1.52 to 3.21) and lower for the later implementation period (aHR 0.71, 95% CI 0.54 to 0.92). Under Treat‐All only, the hazard was higher for pregnant women (aHR 1.37, 95% CI 1.10 to 1.71), WHO staging III/IV (aHR 1.41, 95% CI 1.05 to 1.90) (vs. WHO stage I) and elevated creatinine ≥121 µmol/L (aHR 1.73, 95% CI 1.00 to 2.99).

In supplementary analysis, including health facility as a co‐variate instead of primary versus secondary care level, two primary care facilities under Treat‐All and one primary care facility under SOC showed an increased hazard of an unfavourable outcome when compared with the secondary care facility at each health zone (Figure S5).

## 4 Discussion

This study assessed programmatic and patient outcomes of universal ART provision (Treat‐All) in a predominantly rural public sector setting in Eswatini. Compared to SOC, Treat‐All resulted in comparable retention, improved viral suppression and comparable composite outcomes of retention without viral failure, after adjusting for differences between patients accessing each service. Although crudely a higher proportion of patients were lost to care under Treat‐All, this service also enrolled more patients with higher CD4 counts 36, suggesting improved coverage of the overall HIV population.

### 4.1 Explanation of findings

Similarly to other settings 46, low CD4 cell count was associated with an adverse outcome. The association was more pronounced for patients presenting with advanced HIV disease, and the effect of CD4 cell count varied with time under SOC, with higher hazard early during treatment. Notably, under Treat‐All, outcomes for patients with high CD4 cell counts were similar to those for patients with 201 to 350 cells/mm^3^, a pattern confirmed by two other Treat‐All trials 31, 32 but in contrast to findings from the Western Cape, South Africa, where attrition was increased for CD4 >500 cells/mm^3^ 33.

Overall, the median CD4 cell count at ART initiation was only slightly higher under Treat‐All (37 cells/mm^3^), possibly explained by concurrent expansion of treatment eligibility criteria under SOC during the study period. Pregnant and lactating women were already eligible for prompt ART (PMTCTB+) under SOC and treatment eligibility for non‐pregnant adults was expanded from ≤350 to ≤500 cell/mm^3^. Restricting analysis to non‐pregnant adults under period‐1 (WHO 2010 treatment guideline implementation), the difference in median CD4 cell count increased to 73.5 cells/mm^3^.

ART initiation on the same day as HIV care enrolment was associated with an adverse outcome, possibly stronger during the first year of treatment. Data on same‐day ART initiation remain conflicting, with randomized controlled trials showing benefits and observational studies indicating no benefit or increased risk of unfavourable treatment outcomes 46, 47, 48, 49, 50. A reason could be that observational studies may not be able to sufficiently adjust for time‐dependent confounding. For instance, patients with higher CD4 cell count may be less likely to initiate ART the same day and more likely to have a favourable outcome while immunocompromised patients may be more likely to start treatment on the same day and to have an unfavourable outcome. Patients initiating ART are a subset of those diagnosed, linked and enrolled into care, thus our findings not comparable with studies with follow‐up starting at the time of HIV diagnosis. Further studies are needed to understand same‐day ART and its impact on HIV programmes implementing Treat‐All, specifically because many RLS already apply rapid treatment initiation 3. Nevertheless, attention is needed to identify patients not ready for same‐day ART and to provide adequate adherence support after same‐day ART initiation 51.

Men and non‐pregnant women had the same risk of an unfavourable outcome. Although men in general show worse HIV care outcomes 46, 52, 53, findings from Eswatini remain inconsistent, with increased and similar risk for men 54, 55, 56. Adverse treatment outcomes were high for pregnant women under Treat‐All, which is in line with findings from PMTCT B+ and general ART programmes 46, 48, 57. Specific interventions supporting pregnant women under Treat‐All may be needed to achieve the full benefits of universal ART expansion for this group.

Similar to other studies 37, 48, 58, 59, 60, younger age, being unmarried and clinical factors (BMI, haemoglobin, creatinine) increased the risk of adverse outcomes in both health zones and irrespective of disease progression. In contrast to another setting 58, the level of education did not show associations. While this setting showed significant variations for ART initiation across facilities 36, the variations with respect to programmatic outcomes were minor.

The later WHO 2013 guideline implementation period (time period‐2) showed a lower risk of an unfavourable outcome under SOC. Temporal trends have also been reported from other settings 16, 17, 18, 19, 20, 21, 22. In our case, quality of follow‐up care may be one explanation. This time period coincided with the expansion of differentiated community‐centred ART care models for patients stable on ART and was more pronounced under SOC, which may have supported long‐term adherence and decongested busy facilities 61.

WHO emphasizes that patients in greatest need of ART should not be de‐prioritized during treatment scale‐up 1. Although about one third of patients in both health zones presented with advanced HIV disease and had an increased likelihood of adverse outcome if CD4 was ≤100 cells/mm^3^, covariate‐adjusted analysis indicated that the risk was similar under Treat‐All and SOC. In addition, TB co‐infection emerged as a protective factor during early treatment. As per national guideline recommendations, co‐infected patients may have received more attention by health workers given their high risk of mortality, resulting in less loss to care. Although median CD4 cell count increased during ART programme expansion internationally 62, the challenge of advanced HIV disease is likely to persist 63. Scale‐up of optimized packages of care for patients with advanced HIV (e.g. better diagnostics and effective prophylactic treatment) is essential to further reduce mortality and morbidity 63, 64. Our findings are encouraging in that patients with advanced HIV disease were probably not de‐prioritized under Treat‐All compared with SOC.

### 4.2 Findings in context

Overall retention was comparable to ART programmes in low‐ and middle‐income countries 65 and two Treat‐All trials in Southern Africa 32, 33. However, point estimates of retention tended to be lower than under SOC, than previous retention estimates from this setting before the introduction of Treat‐All 38 and than in a streamlined combination intervention trial in Eastern Africa 31. Similarly to another Treat‐All trial in South Africa 32, 6% of patients never returned for a clinic visit after ART initiation (vs. 3% under SOC). A broad range of supportive interventions may improve retention (e.g. community‐based adherence support, health technology interventions) 66, 67, 68, 69, potentially also under Treat‐All 27.

While VL testing uptake was low and delayed in both health zones, crude viral suppression tended to be slightly higher under Treat‐All and comparable to other settings 70. Overall, viral failure seemed lower than in other ART programmes 71, 72, possibly explained by variability of definitions, underestimation of true viral failure because of suboptimal VL testing coverage and record keeping, and high viral re‐suppression rates (~60%) in patients with single elevated VLs 43, 73.

### 4.3 Limitations and strengths

First, our estimates of ART retention are conservative. Previous studies showed that transient treatment interruptions and movements between clinics are common and many patients recorded with LTFU are retained 26, 74, 75. Because of limitation in routine monitoring and limited tracking of patients lost to follow‐up, this study was not able to adjust for silent transfer between treatment sites and silent return to care. In addition, ART retention in clinic was measured rather than retention in care or national‐level retention, likely biasing estimation of retention downwards 76, 77. We also did not report on overall HIV care retention of patients entering care as done in other routine Treat‐All settings 37, thus possibly not detecting a higher care retention benefit of Treat‐All when compared with SOC. Finally, not accounting for transient treatment interruptions possibly introduced a spurious trend of increased LTFU in our cohort, which had a relatively short follow‐up time (analysis bias) compared with other cohorts 78. Second, given the observational study design and comparison of two different health zones, we may not have been able to adjust for all unobserved variables (e.g. exposure to differentiated service delivery model for patients stable on ART). Third, assessing ART coverage and population‐level viral suppression due to Treat‐All was beyond the scope of this analysis. Nevertheless, ART initiation rates measured from the time of facility‐based HIV care enrolment was higher under Treat‐All (91%) than SOC (74%; *p* < 0.001) in this setting 36. This possible additional ART coverage under Treat‐All may have an increased overall effect on viral suppression of the entire population living with HIV despite lower retention in crude analysis. In addition, ART has been progressively expanded in this setting since 2006 38, achieving 82.7% population‐level ART coverage and 79.1% population‐level VL suppression among people living with HIV in 2016/17 79.

Despite the wide‐scale adoption of Treat‐All in RLS 5, 80, studies accounting for this policy change under routine conditions are lacking. This study began two years before publication of the WHO Treat‐All guidelines, and thus has the potential to inform implementation of this policy in similar rural settings. We adjusted for a wide range of covariates, which likely enabled us to show a comprehensive picture of Treat‐All. In addition, we encountered risk factors that have not been widely described previously (e.g. same‐day ART initiation) but that may affect programmatic outcomes of large HIV programmes. Finally, we assessed the programmatic impact of treatment expansion on patients with advanced HIV disease.

## 5 Conclusions

Compared to SOC, Treat‐All resulted in comparable retention, improved viral suppression and comparable composite outcomes of retention without viral failure. Patients with advanced HIV disease were possibly not de‐prioritized and predictors of unfavourable outcomes were comparable between Treat‐All and SOC. This study contributes to evidence that treatment expansion through the Treat‐All programmatic approach may be feasible in RLS without increasing unfavourable outcomes, and as such is likely to have public health benefits.

## Competing Interest

The authors declare no conflict of interest.

## Authors' contributions

BK, KJ and RT designed the study. BK, KJ and SMK established the cohort and were involved in data acquisition. BK, MS, AB and IC led the data analysis plan and SMK, EM, SMH and BR contributed to the analysis. BK performed the statistical analyses and wrote the first draft of the manuscript. MS, AB and IC advised on final analyses. BK, MS, KJ, SMK, RT, EM, SMH, BR, IC and AB interpreted the data, contributed to the writing of the manuscript and approved the final version.

## Abbreviations

aHR, Adjusted hazard ratio; ALT, Alanine aminotransferase; ART, Antiretroviral therapy/treatment; BMI, Body mass index; CI, Confidence interval; EFV, Efavirenz; IQR, Interquartile range; LTFU, Loss to follow‐up; PMTCT B+; Prevention of mother‐to‐child transmission option B+; RLS, Resource‐limited setting; SOC, Standard of care; TB, Tuberculosis; TDF, Tenofovir disoproxil fumarate; VL, Viral load; WHO, World Health Organization.

## Supporting information


**Table S1.** Complete and missing values for covariate and imputation procedures.
**Table S2.** Distribution of CD4 cell count and WHO clinical staging for patients with advanced IV disease under Treat‐All and SOC (n = 1060).
**Table S3.** Kaplan‐Meier estimates of retention under Treat‐All for selected variables.
**Table S4.** Predictors of the unfavourable outcome for the entire cohort (Treat‐All and SOC combined) initiated on first‐line ART (n = 3170).
**Figure S1.** Trace plots of imputed data for all covariates with missing values.
**Figure S2.** Kernel density plots for imputed haemoglobin for all imputed datasets as an example using the *midiagplots* command in Stata.
**Figure S3.** Kaplan‐Meier graphs of retention under Treat‐All for selected variables.
**Figure S4.** Absolute difference in hazard of an unfavourable outcome by TB status for the entire cohort (Treat‐All and SOC combined).
**Figure S5.** Variations in adjusted hazard ratios of the composite unfavourable outcome comparing primary care facilities with the secondary care facility under Treat‐All (facility 1) and under standard of care (facility 10).
