# Supplementary material for: HIV programmatic outcomes following implementation of the ‘Treat‐All’ policy in a public sector setting in Eswatini: a prospective cohort study
Source: J Int AIDS Soc. 2020 Mar 3;23(3):e25458. doi: 10.1002/jia2.25458 (PMC7054447; doi:10.1002/jia2.25458)
Supplement: Supplementary file 1 — Table S1. Complete and missing values for covariate and imputation procedures. Table S2. Distribution of CD4 cell count and WHO clinical staging for patients with advanced IV disease under Treat‐All and SOC (n = 1060). Table S3. Kaplan‐Meier estimates of retention under Treat‐All for selected variables. Table S4. Predictors of the unfavourable outcome for the entire cohort (Treat‐All and SOC combined) initiated on first‐line ART (n = 3170). Figure S1. Trace plots of imputed data for all covariates with missing values. Figure S2. Kernel density plots for imputed haemoglobin for all imputed datasets as an example using the midiagplots command in Stata. Figure S3. Kaplan‐Meier graphs of retention under Treat‐All for selected variables. Figure S4. Absolute difference in hazard of an unfavourable outcome by TB status for the entire cohort (Treat‐All and SOC combined). Figure S5. Variations in adjusted hazard ratios of the composite unfavourable outcome comparing primary care facilities with the secondary care facility under Treat‐All (facility 1) and under standard of care (facility 10). [file JIA2-23-e25458-s001.docx]

**Supplementary material**

Study:

**HIV programmatic outcomes following implementation of the ‘Treat-All’ policy in a public sector setting in Eswatini: a prospective cohort study**

*Bernhard Kerschberger^1,2^, Michael Schomaker^2,3^, Kiran Jobanputra^4^, Serge M Kabore^1^, Roger Teck^4^, Edwin Mabhena^1^, Simangele Mthethwa-Hleza^5^, Barbara Rusch^6^, Iza Ciglenecki^6^, Andrew Boulle^2^*

*1 Médecins Sans Frontières (Operational Centre Geneva), Mbabane, Eswatini*

*2 Centre for Infectious Disease Epidemiology and Research, School of Public Health and Family Medicine, University of Cape Town, Cape Town, South Africa*

*3 Institute of Public Health, Medical Decision Making and HealthTechnology Assessment, UMIT - University for Health Sciences, Medical Informatics and Technology, Hall in Tirol, Austria*

*4 The Manson Unit, Médecins Sans Frontières, London, United Kingdom*

*5 Eswatini National AIDS Programme (ENAP), Ministry of Health, Mbabane, Eswatini*

*6 Médecins Sans Frontières (Operational Centre Geneva), Geneva, Switzerland*

1. **Missing values and imputation diagnostics**

**Table S1.** Complete and missing values for covariate and imputation procedures.

|  | **Complete observations (n)** | **Missing values (%)** |
| --- | --- | --- |
| **Time since HIV diagnosis** | 3155 | 0.5% |
| **Pregnancy** | 3154 | 0.5% |
| **Marital status** | 3125 | 1.4% |
| **Education** | 2715 | 14.4% |
| **CD4 count** | 3075 | 3.0% |
| **WHO clinical stage** | 3154 | 0.5% |
| **BMI** | 2971 | 6.3% |
| **Haemoglobin** | 2452 | 22.6% |
| **ALT** | 2216 | 30.1% |
| **Creatinine** | 2516 | 20.6% |
| **Phone availability** | 3137 | 1.0% |

*ALT, alanine aminotransferase; BMI, body mass index; WHO, World Health Organization.*

*Missing values were assumed to be missing at random. Multivariate imputation by chained equations was used, as implemented in Stata 14.1., to imput missing values in 20 datasets, using predictive mean matching for continues variables, ordered logistic regression for ordinal variables, multinomial logistic regression for nominal variables and logistic regression for binary variables [1]. Imputation diagnostic was performed after imputation by plotting trace plots and midiagplots [2].*

*1. mi impute chained — Impute missing values using chained equation [Internet]. Stata. [cited 2019 Jan 25]. Available from: https://www.stata.com/manuals13/mimiimputechained.pdf*

*2. Eddings W, Marchenko Y. Diagnostics for multiple imputation in Stata. Stata J. 2012;12(3):353–67.*


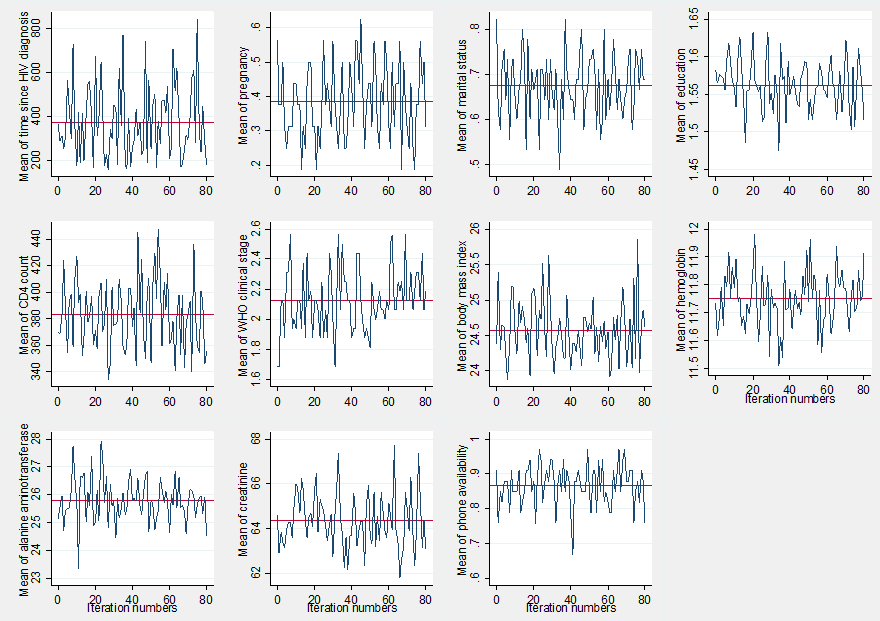


**Figure S1.** Trace plots of imputed data for all covariates with missing values.


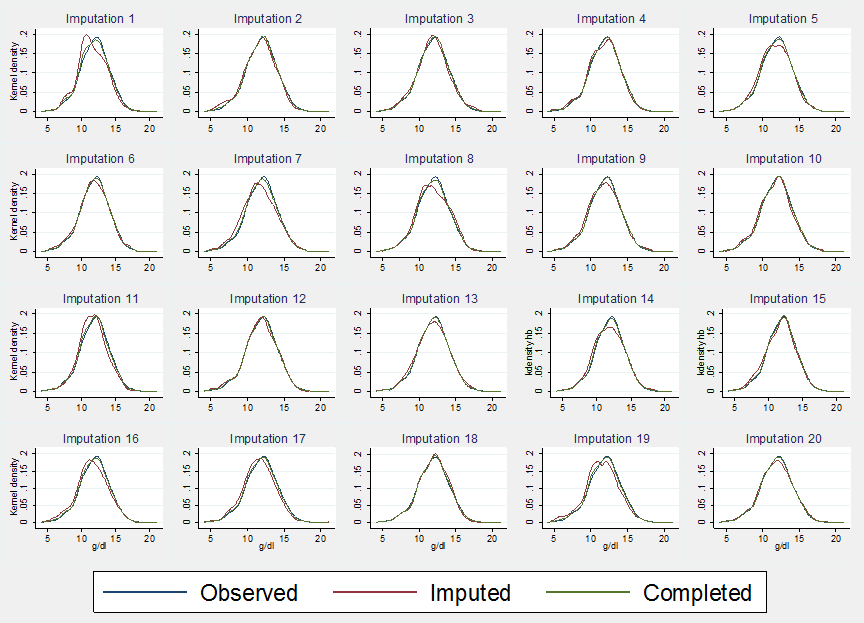


**Figure S2.** Kernel density plots for imputed haemoglobin for all imputed datasets as an example using the *midiagplots* command in Stata.

1. **Additional results**

**Table S2:** Distribution of CD4 cell count and WHO clinical staging for patients with advanced HIV disease under Treat-All and SOC (n=1060).

|  |  | **CD4 cell count in cells/mm^3^** | | | | |
| --- | --- | --- | --- | --- | --- | --- |
|  |  | **0–100** | **101–200** | **201–350** | **351–500** | **≥501** |
| **WHO clinical staging** | **I** | 152 (32.7) | 285 (60.6) | 0 (0) | 0 (0) | 0 (0) |
|  | **II** | 113 (24.3) | 92 (19.6) | 0 (0) | 0 (0) | 0 (0) |
|  | **III/IV** | 200 (43.0) | 93 (19.8) | 68 (100) | 29 (100) | 28 (100) |

Advanced HIV disease is *defined as CD4 <200 cells/mm3 and/or WHO III/IV clinical staging.*

*Patients with missing CD4 cell count and/or WHO clinical staging are not presented in the table (n=14).*

**Table S3:** Kaplan–Meier estimates of retention under Treat-All for selected variables.

|  | **6 months** | **12 months** | **24 months** | **36 months** |
| --- | --- | --- | --- | --- |
| **Sex-pregnancy status** |  |  |  |  |
| Men | 87 (84 to 90) | 81 (77 to 84) | 75 (71 to 79) | 72 (68 to 76) |
| Non-pregnant women | 85 (83 to 87) | 80 (78 to 83) | 73 (70 to 76) | 70 (67 to 74) |
| Pregnant women | 77 (73 to 81) | 69 (64 to 73) | 61 (56 to 65) | 60 (55 to 64) |
| **Age at HIV care enrolment, years** |  |  |  |  |
| 16–24 | 78 (74 to 82) | 69 (65 to 73) | 60 (55 to 64) | 58 (53 to 63) |
| 25–49 | 85 (83 to 87) | 80 (78 to 82) | 73 (71 to 75) | 71 (68 to 73) |
| ≥50 | 86 (80 to 91) | 82 (76 to 88) | 80 (73 to 85) | 76 (68 to 82) |
| **CD4 count, cells/mm^3^** |  |  |  |  |
| 0–100 | 80 (75 to 85) | 72 (66 to 77) | 64 (58 to 70) | 59 (52 to 65) |
| 101–200 | 83 (78 to 87) | 77 (72 to 82) | 70 (64 to 75) | 67 (61 to 73) |
| 201–350 | 86 (82 to 89) | 80 (76 to 84) | 73 (69 to 77) | 70 (63 to 76) |
| ≥351 | 85 (83 to 87) | 79 (76 to 82) | 72 (69 to 75) | 71 (68 to 74) |

**Table S4**: Predictors of the unfavourable outcome for the entire cohort (Treat-All and SOC combined) initiated on first-line ART (n = 3170).

|  | **Univariate analysis** | | **Multivariate analysis^5^** | |
| --- | --- | --- | --- | --- |
|  | **HR** | **95% CI** | **aHR** | **95% CI** |
| **Health zone** |  |  |  |  |
| SOC | 1 |  | 1 |  |
| Treat-All | 1.21 | (1.06 to 1.39) | 1.04 | (0.90 to 1.20) |
| **Implementation period^1^** |  |  |  |  |
| Period-1 | 1 |  | 1 |  |
| Period-2 | 1.02 | (0.88 to 1.18) | 0.93 | (0.80 to 1.08) |
| **Facility** |  |  |  |  |
| PHC | 1 |  | 1 |  |
| SHC^2^ | 0.96 | (0.84 to 1.10) | 0.88 | (0.76 to 1.03) |
| **Time since HIV diagnosis^3^** |  |  |  |  |
| ≥90 days | 1 |  | 1 |  |
| 1–89 days | 1.60 | (1.36 to 1.88) | 1.10 | (0.89 to 1.35) |
| Same day | 2.03 | (1.68 to 2.45) | 0.95 | (0.75 to 1.21) |
| **Time since HIV care enrolment^4^** |  |  |  |  |
| ≥90 days | 1 |  | 1 |  |
| 1–89 days | 2.13 | (1.69 to 2.67) | 1.74 | (1.29 to 2.33) |
| Same day | 3.03 | (2.42 to 3.80) | 2.38 | (1.79 to 3.17) |
| **Sex** |  |  |  |  |
| Men | 1 |  | 1 |  |
| Women | 1.24 | (1.06 to 1.44) | 1.10 | (0.91 to 1.32) |
| **Pregnancy** |  |  |  |  |
| No | 1 |  | 1 |  |
| Yes | 1.59 | (1.37 to 1.83) | 1.33 | (1.12 to 1.59) |
| **Age at HIV care enrolment, years** |  |  |  |  |
| 16–24 | 1.69 | (1.46 to 1.96) | 1.36 | (1.15 to 1.61) |
| 25–49 | 1 |  | 1 |  |
| ≥50 | 0.84 | (0.64 to 1.10) | 0.99 | (0.75 to 1.32) |
| **Marital status** |  |  |  |  |
| Married | 1 |  | 1 |  |
| Not married | 1.69 | (1.46 to 1.96) | 1.44 | (1.23 to 1.68) |
| **Education** |  |  |  |  |
| None | 1 |  | 1 |  |
| Primary | 1.04 | (0.75 to 1.45) | 1.00 | (0.71 to 1.41) |
| Secondary | 1.19 | (0.87 to 1.63) | 1.00 | (0.71 to 1.40) |
| Tertiary | 1.65 | (0.95 to 2.87) | 1.47 | (0.83 to 2.63) |
| **CD4 count, cells/mm^3^** |  |  |  |  |
| 0–100 | 1.67 | (1.36 to 2.05) | 1.57 | (1.25 to 1.98) |
| 101–200 | 1.27 | (1.02 to 1.59) | 1.27 | (1.01 to 1.59) |
| 201–350 | 1 |  | 1 |  |
| 351–500 | 1.12 | (0.92 to 1.38) | 1.21 | (0.98 to 1.48) |
| ≥501 | 1.21 | (0.98 to 1.48) | 1.22 | (0.99 to 1.52) |
| **WHO clinical stage** |  |  |  |  |
| I | 1 |  | 1 |  |
| II | 1.00 | (0.84 to 1.20) | 1.02 | (0.84 to 1.23) |
| III/IV | 1.35 | (1.12 to 1.61) | 1.28 | (1.02 to 1.61) |
| **Tuberculosis** |  |  |  |  |
| No | 1 |  | 1 |  |
| Yes | 0.94 | (0.73 to 1.22) | 0.62 | (0.45 to 0.84) |
| **BMI, kg/m^2^** |  |  |  |  |
| <18.5 | 1.71 | (1.36 to 2.15) | 1.63 | (1.27 to 2.09) |
| 18.5–24.9 | 1 |  | 1 |  |
| ≥25 | 0.87 | (0.76 to 1.01) | 0.89 | (0.76 to 1.04) |
| **Haemoglobin, g/dL** |  |  |  |  |
| ≤9 | 1.72 | (1.46 to 2.03) | 1.34 | (1.12 to 1.61) |
| ≥10 | 1 |  | 1 |  |
| **ALT, U/L** |  |  |  |  |
| ≤42 | 1 |  | 1 |  |
| ≥43 | 0.81 | (0.64 to 1.03) | 0.85 | (0.66 to 1.10) |
| **Creatinine, µmol/L** |  |  |  |  |
| ≤120 | 1 |  | 1 |  |
| ≥121 | 1.65 | (1.08 to 2.52) | 1.62 | (1.04 to 2.52) |
| **NRTI** |  |  |  |  |
| TDF | 1 |  | 1 |  |
| AZT | 1.01 | (0.70 to 1.46) | 1.08 | (0.61 to 1.92) |
| ABC | 1.63 | (0.78 to 3.44) | 1.52 | (0.70 to 3.26) |
| **NNRTI** |  |  |  |  |
| EFV | 1 |  | 1 |  |
| NVP | 0.93 | (0.65 to 1.33) | 1.04 | (0.60 to 1.80) |
| **Phone availability** |  |  |  |  |
| No | 1 |  | 1 |  |
| Yes | 0.92 | (0.74 to 1.14) | 0.88 | (0.70 to 1.10) |

*ABC, abacavir; aHR, adjusted hazard ratio; ALT, alanine aminotransferase; AZT, zidovudine; BMI, body mass index; CI, confidence interval; EFV, efavirenz; HR, hazard ratio; NNRTI, non-nucleoside reverse transcriptase inhibitors; NRTI, nucleoside reverse transcriptase inhibitor; NVP, nevirapine; PHC, primary healthcare level; SHC, secondary healthcare level; SOC, standard of care; TDF, tenofovir disoproxil fumarate; WHO, World Health Organization.*

*^1^ Period-1 is the WHO 2010 (from October 2014 to October 2015) and period-2 is the WHO 2013 (from November 2015 onwards) ART initiation guideline implementation period as followed under standard of care.*

*^2^ Secondary healthcare: ART outpatient departments in one health centre (with inpatient capacity) in Treat-All and ART outpatient departments in one hospital in standard of care.*

*^3^ This is the time from HIV diagnosis to ART initiation.*

*^4^ This is the time from facility-based HIV care enrolment to ART initiation.^5^ The flexible parametric model had four internal knots and one internal knot for the time-varying covariate (TB case).*


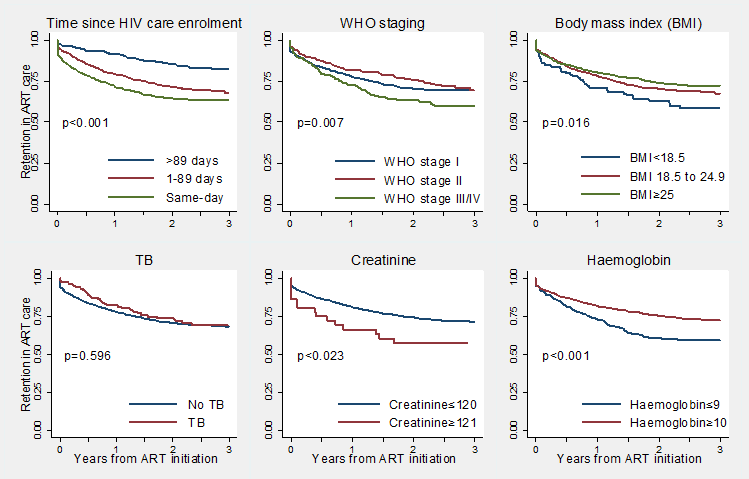


**Figure S3**. Kaplan–Meier graphs of retention under Treat-All for selected variables.


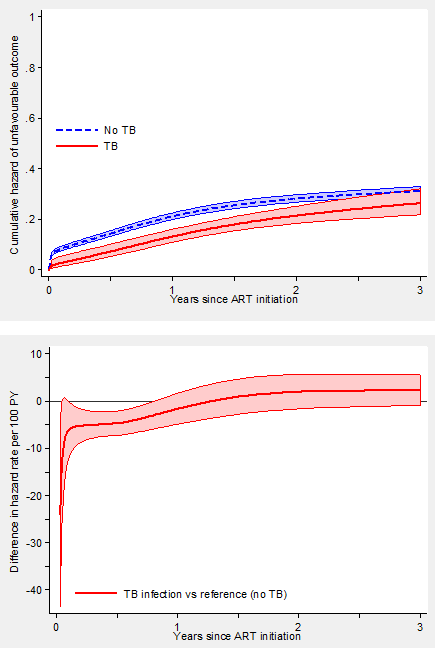


*ART, antiretroviral therapy; PY, person years.*

*The effect of baseline TB varied over time, with lower hazard of an unfavourable outcome during the first 8 months of TB treatment and similar hazard thereafter.*

**Figure S4.** Absolute difference in hazard of an unfavourable outcome by TB status for the entire cohort (Treat-All and SOC combined).


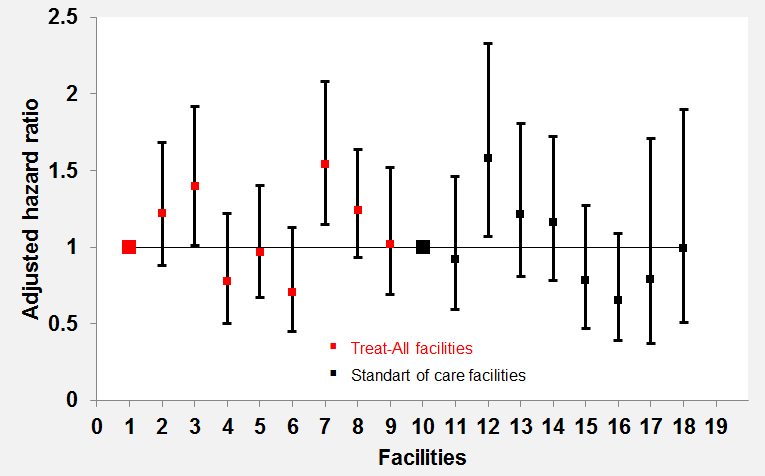


**Figure S5:** Variations in adjusted hazard ratios of the composite unfavourable outcome comparing primary care facilities with the secondary care facility under Treat-All (facility 1) and under standard of care (facility 10).
